# Extracellular vesicles in urologic malignancies—Implementations for future cancer care

**DOI:** 10.1111/cpr.12659

**Published:** 2019-08-30

**Authors:** Zhangsong Wu, Zhiqiang Zhang, Wuchao Xia, Jiajia Cai, Yuqing Li, Song Wu

**Affiliations:** ^1^ Medical College Shenzhen University Shenzhen China; ^2^ Department of Urological Surgery, The Third Affiliated Hospital of Shenzhen University Shenzhen University Shenzhen China; ^3^ Shenzhen Following Precision Medical Institute, The Third Affiliated Hospital of Shenzhen University Shenzhen University Shenzhen China; ^4^ Medical College Anhui University of Science and Technology Huainan China; ^5^ Department of Urological Surgery, The First Affiliated Hospital of Guangzhou Medical University Guangzhou Medical University Guangzhou China

**Keywords:** bladder cancer, exosomes, extracellular vesicles, kidney cancer, microvesicles, prostate cancer

## Abstract

Extracellular vesicles (EVs), a heterogeneous group of vesicles differing in size and shape, cargo content and function, are membrane‐bound and nano‐sized vesicles that could be released by nearly all variations of cells. EVs have gained considerable attention in the past decades for their functions in modulating intercellular signalling and roles as potential pools for the novel diagnostic and prognostic biomarkers, as well as therapeutic targets in several cancers including urological neoplasms. In general, human and animal cells both can release distinct types of EVs, including exosomes, microvesicles, oncosomes and large oncosomes, and apoptotic bodies, while the content of EVs can be divided into proteins, lipids and nucleic acids. However, the lack of standard methods for isolation and detection platforms rein the widespread usage in clinical applications warranted furthermore investigations in the development of reliable, specific and sensitive isolation techniques. Whether and how the EVs work has become pertinent issues. With the aid of high‐throughput proteomics or genomics methods, a fully understanding of contents contained in EVs from urogenital tumours, beyond all doubt, will improve our ability to identify the complex genomic alterations in the process of cancer and, in turn, contribute to detect potential therapeutic target and then provide personalization strategy for patient.

## 1 INTRODUCTION

Urogenital carcinomas are mainly leading to morbidity and mortality worldwide.^1^ Although therapeutic strategies (surgery, biotherapy and chemotherapy) for patients suffering from urinary cancers have improved, the curative and monitoring efficacy for these cancers still remains poor, as existing tests (general examinations and biopsies) are not a sufficiently sensitive or specific and heterogeneous peculiarity of these malignancies.^2^ Moreover, the deep location of urogenital cancers makes them hard to access to be diagnosed at early stages. Crosstalk between cells and their microenvironment is a fundamental principle under the normal and pathological condition.^3^ EVs, small membrane‐bound vesicles, serve as important players of bidirectional communication,^4^, ^5^ which released from almost eukaryotic and prokaryotic cells with transmitting complicate messages from donor cells towards anchored cells, and have been discovered in various types of body fluids including urine, blood and bile.^6^ According to the ways of production and secretion, EVs are produced by inward budding of intercellular endosomes, which are defined as exosomes (40‐1200 nm), EVs straightly shed by budding from the cell membrane, which are almost recognized as large oncosomes or microvesicles, with 1‐10 μm or 50‐1500 nm, and apoptotic vesicles are released during cell undergoing apoptosis ranged from 50 to 2000 nm, respectively.^7^, ^8^, ^9^

Extracellular vesicles serve as an appealing source for the development of biomarkers as their membrane‐bound structure to protect against exogenous proteases and RNases.^10^, ^11^ The biological function of EVs is performed by cytosolic lipids, proteins, DNA, mRNA, miRNA, lincRNA and other non‐coding RNAs, as well as cell membrane.^12^ In addition, cancer cell releases more EVs than that normal one does.^13^ Herein, we introduce EVs briefly and provide a comprehensive overview of their biophysical properties, roles and applications in the most common urologic neoplasms, including kidney, prostate and bladder, and discuss potential clinical applications in the future.

## 2 EV CLASSES, BIOGENESIS AND CONTENTS

**Figure 1:**
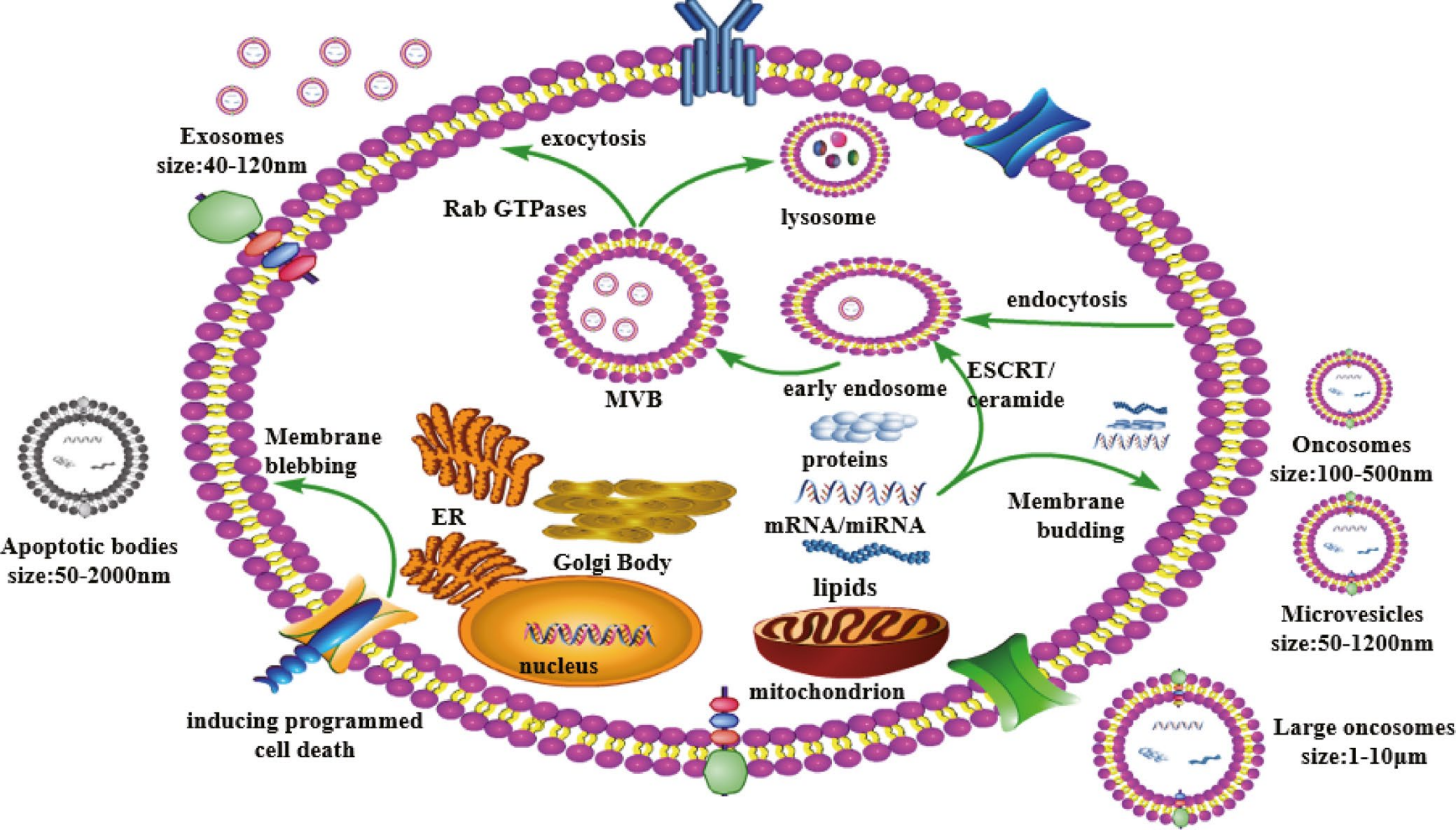
Release and uptake mechanisms of extracellular vesicles. Extracellular vesicles can be classed as exosomes, microvesicles and apoptotic bodies, based on the mechanism by which they are released from cells and differentiated based on their size and content. MVs, and oncosomes or large oncosomes are directly shed or bud from the plasma membrane. Apoptotic bodies are released from the cell undergoing programmed cell death. Exosomes are formed by inward budding of multivesicular bodies

Our current understanding of EVs indicates that at least four heterogeneous types of EVs have been identified based on their mechanism of formation and distinguished size: microvesicles, exosomes, oncosomes or large oncosomes, and apoptotic bodies (Table 1). In general, the formation of exosomes and microvesicles is two completely different approaches, but they function similarly. Oncosomes or large oncosomes resemble the way of microvesicles via membrane budding. Apoptotic bodies specifically arise resulting in indiscriminate membrane bubbling during apoptosis (Figure 1).

**Table 1:** Details of different extracellular vesicles

	Exosomes	Microvesicles	Oncosomes	Large oncosomes	Apoptotic bodies
Size	40‐120 nm	50‐1500 nm	100‐500 nm	1‐10 μm	50‐2000 nm
Intracellular origin	Endosomes	Plasma membrane	Plasma membrane	Plasma membrane	Plasma membrane
Electron microscopy	Round shape	Irregular shape	Irregular shape	Amoeboid phenotype	Heterogenous
Release	Endolysosomal pathway, internal budding, exocytosis	Membrane budding	Membrane budding	Membrane budding	Generated as a result of apoptotic disintegration, resulting vesicles become part of the extracellular milieu
Marker proteins	Membrane‐associated proteins: tetraspanin (CD9, CD63, CD81, CD82). Endosomal sorting complex required for Transport‐associate protein: Tsg101, ALIX. Cytoplasmic proteins: Hsp70, Hsp90. Membrane transport and fusion proteins: Rab GTPases, annexins	Integrins, selectins, CD40 ligand	Integrins, selectins, Membrane‐associated proteins	Integrins, selectins, VDAC ½ SLC25A3/5/6 ITGA5/6	Histones, C3b, Annexin V, Caspase 3
Contents	Proteins, lipids, mRNA, miRNA and cytosol	Proteins, lipids, mRNA, miRNA and cytosol	Proteins, lipids, mRNA, miRNA and cytosol	Proteins, lipids, mRNA, miRNA and cytosol	Proteins, lipids, DNA, rRNA, organelles and cytosol

Abbreviations: C3b, complement component 3; HSP, heat shock proteins; ITGA5/6, human integrin alpha 5/6; miRNA, microRNA; SLC25A3/5/6, solute carrier family 25 member3/5/6; VDACs, Voltage‐dependent anion channels.

Exosomes generally form an early endosome by the endocytosis and internalization of cell‐surface receptors into membrane‐bound vacuoles in the first step,^14^ which then matures to generate a late endosome within undergoing several changes, such as the limiting membrane of the late endosome then buds inward and pinches off as the result of the formation of intraluminal vesicles (ILVs), also known as multivesicular bodies, and ILVs traffick to and fuse with the plasma membrane leading in releasing exosomes eventually.^15^ ESCRT‐0‐III plays significant roles in driving exosome formation^16^, ^17^; in addition, multivesicular bodies could intermediate in the lysosomal degradation pathway.^18^ However, the mechanisms related to the fusion of multivesicular bodies with the cellular membrane are uncovered, which may be regulated by several factors including lipid ceramide and Rab GTPase (including Rab5 and Rab7) proteins and ESCRT.^19^, ^20^, ^21^ Numerous literatures have indicated that several biomarkers expressed in the exosome differentially compared with another types of EVs, including heat shock proteins (eg HSP60, 70 and 90), tetraspanins (eg CD9, CD63 and CD81), membrane transporters, fusion proteins, ALG‐2‐interacting protein X (Alix) and tumour susceptibility gene 101 protein (TSG101).^20^, ^22^

In contrast, microvesicles with nano‐sized with 100‐1500 nm are straightforwardly shed from the cellular membrane responding to stimuli or physiological conditions.^23^ It is believed that ADP ribosylation factor 6 (ARF6) can meditate the freeing of protease‐loaded vesicles from the cellular membrane due to the crosstalk with Rho signalling pathways.^24^, ^25^, ^26^ Moreover, microvesicles are specifically produced in the cellular membrane regions that are linked to be enriched in cholesterol, ceramide and lipid rafts.^27^ TSG101 is also known to interact with accessory proteins Alix and arresting domain‐containing protein‐1 (ARRDC1) during releasing microvesicles, illustrating that microvesicles sharing some same characteristics with exosomal biogenesis.^28^ As described for microvesicles, oncosomes and large oncosomes are generated by plasma membrane budding, with amoeboid‐like phenotype. Notably, oncosomes and large oncosomes specially derived from cancerous cells are indicated to play vital roles in malignancies invasion. The term “oncosome” is firstly referred that the EVs with size range from 100 to 500 nm. Subsequently, large non‐apoptotic EVs were detected in prostate tumours with their unusual size in 1‐10 mm, called large oncosomes. Currently, some studies demonstrate that oncosomes and large oncosomes are definitely different variety of EVs respecting to their size, cargo contents and target effects, and additional studies are therefore of the essence to clarify differences between oncosomes and large oncosomes.

Apoptotic bodies, imposing an effect on the cellular response by transmitting their substance towards receptor cells, are generated during undergoing programmed cell death with 500‐4000 nm, and their content contains fragmented cytoplasmic organelles as well as destructive nuclei.^29^, ^30^

## 3 ISOLATION TECHNIQUES OF EVS

**Figure 2:**
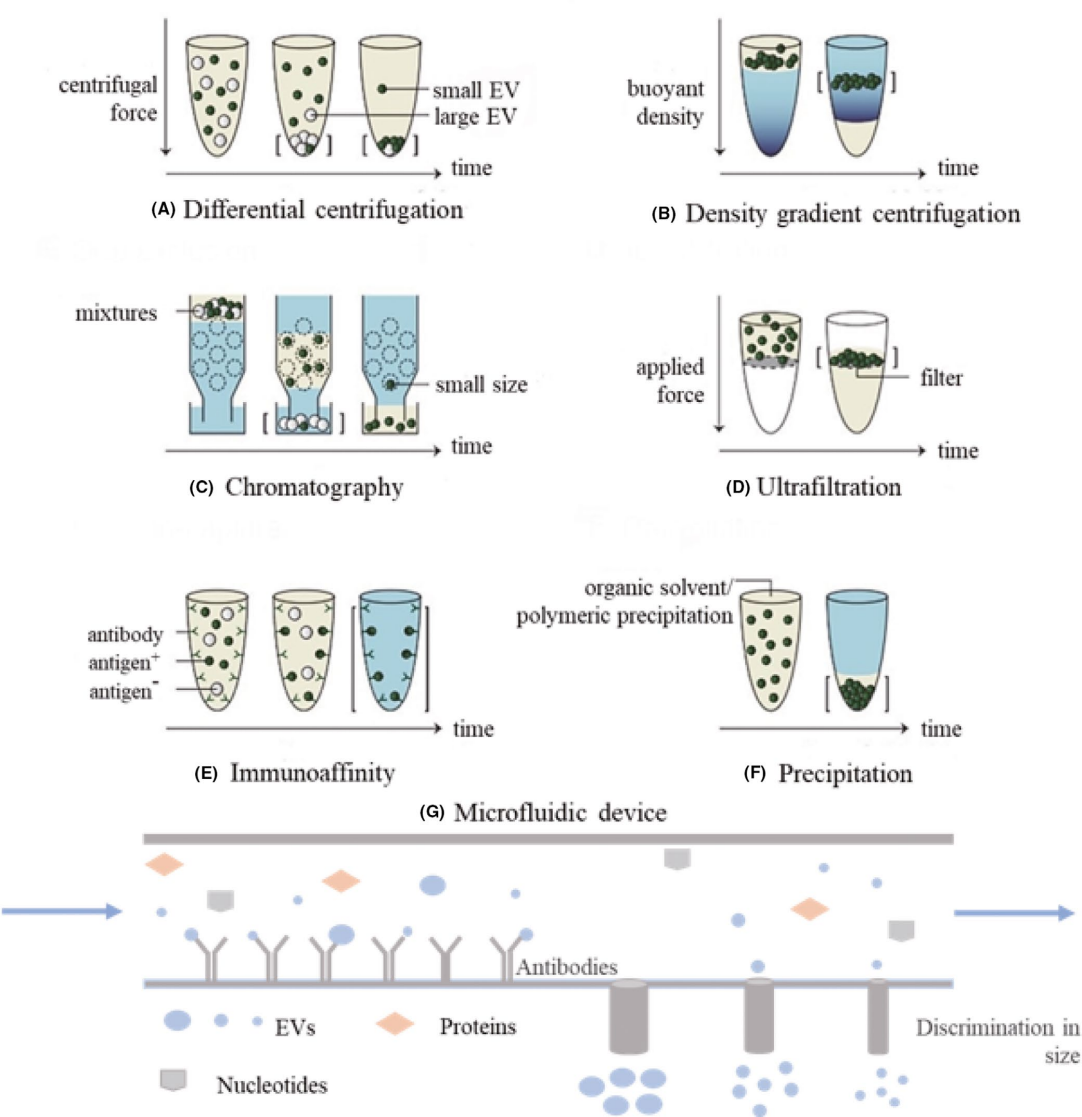
The common methods to isolate EVs. Straight brackets: isolated EVs; yellow: soluble components; and blue: buffer. A, In differential centrifugation, separation is based on sedimentation velocity, largely depended by size; B, in density gradient centrifugation, separation is relied on buoyant and density; C, size exclusion chromatography uses a porous matrix (dotted circles) that separates on size; D, in ultrafiltration, separation is based on size; E, in immunocapture assays, EVs are captured based on the presence of specific EVs surface molecules. F, in precipitation, EVs isolation via adding some water‐excluding polymers to sample to force the precipitation of small EVs out. G, In microfluidic device, EVs isolation via combining several methods such as immunoaffinity and filtration systems. Copyright 2017, University of Helsinki, Frank AW Coumans^31^

No remarkable consensus is in existence of the best approach for isolation, qualitative and quantitative analysis of EVs. There are listing several methods for the isolation of EVs (Figure 2) and demonstrate the available disadvantages and advantages as well (Table 2).^31^

**Table 2:** Summary of EVs isolation techniques

Methods	Isolation method	Isolation Principle	Advantages	Limitations
Centrifugation	Differential centrifugation	Sedimentation velocity	Broad application Standardization Ease of use Reproducibility	High equipment costs, cumbersome long run times and low portability. Time‐consuming. Recovery based on sedimentation efficiency. No absolute separation of vesicle subpopulations. Risk of contamination and formation of protein aggregates
	Density gradient centrifugation	Buoyant density	Lower contamination risks because proteins partition into different density layers than EVs Large sample capacity and yield Good purity and preserved morphology of isolated EVs Unlike sucrose, iodixanol forms iso‐osmotic solutions at all densities, thus better preserving the MV size	Requires expensive ultracentrifuge Time‐consuming Sucrose toxicity might limit downstream functional studies No absolute separation of vesicle subpopulations owing to overlapping density
Filtration	Ultrafiltration	Size	Easy to use Quick technique Reproducible	Small sample volume limitations, Protein contamination Loss of yield owing to trapping in filter pores
	Chromatography	Size/charge	Increases purity and integrity Suitable for isolation from complex biofluids	Requires specialized equipment Small sample volume limitations Time‐consuming
Immunoaffinity	Immunological separation	Presence of specific EVs surface molecules	Isolation of all or specific subtypes of EVs Higher purity of EVs than with UC Possible quantification and characterization of EV protein	Requires prior knowledge of vesicle characteristics Requires specific antibody Not suitable for large sample volumes Captured vesicles might not retain functionality after elution
Precipitation based	Polymeric precipitation	PEG precipitation	High speed Simple procedure High yield	Low purity caused by contamination Low specificity
	Protein organic solvent precipitation	The ion‐pairing effect	Technique overcomes the disadvantages of coextraction of proteins MV denaturation found with highspeed UC Isolation efficiency higher than that of UC	Co‐precipitation of other non‐EV contaminants (proteins, lipoproteins and polymeric materials) Long run times, tedious sample preparation and lengthy pre– and post–clean‐up
Microfluidics based	Microfluidics	Presence of specific molecules, Physical properties such as size, Microfluidic filtration	Increases throughput and allow multiplexing Reduced cost, sample size and processing time	Lack of standardization and clinically applicable methods Microfluidic devices can damage MVs due to shear stress There are scalability and validation issues in clinical practice Drawback of capturing only specific EV populations with IC‐based methods, and low recovery with sieving approaches

Abbreviations: PEG, polyethylene glycol.

## 4 CENTRIFUGATION

In recent, differential centrifugation is the most common technique in responding to isolating EVs, and this approach is consisting of three main centrifugation processes: low speed to eliminate a main portion of the cells, then intermediate speed to subside cell debris and aggregate biopolymers and the other structures with density higher than that of EVs and finally high speed to pellet extracellular vesicles. The advent of density gradient ultracentrifugation increases the efficiency of particle separation according to their buoyant density. However, an important disadvantage of differential centrifugation cannot thoroughly separate protein and other non‐exosomal particles from EVs, limiting its efficacy and usage in clinical studies for diagnosis, to large extent, the advent of density gradient ultracentrifugation that reverses the poor separation efficiency due to their buoyant density, and it is frequently used for EV isolation though with a considerable loss of EVs.^31^, ^32^

## 5 FILTRATION

Due to the micropores or nanopores, EVs can also be isolated by numerous protocols (eg ultrafiltration and hydrostatic dialysis). Ultrafiltration is currently available method used for EV isolation, which represents an efficient alternative to ultracentrifugation. It involves the use of membrane filters with a narrow range of pore size distribution to deplete the proteins with molecular weight over 100 kDa, cell debris and floating cells. However, the size of filters is especially suitable for cell culture media and urine samples, which limit their applications to clinical routine tests. Hydrostatic filtration dialysis (HFD), another approach developed for isolating EVs from urine samples, shows its advantages on the removal of ultracentrifugation via multiple steps and the possibility of isolating EVs from highly diluted solutions. Gel filtration, also called size exclusion chromatography, is one of the methods to collect EVs based on the basis of the size differences, and the disadvantages of this method are its low yield and rather expensive chromatographic sorbent.^33^, ^34^

## 6 PRECIPITATION METHODS

The current research provides numerous protocols for EV isolation via adding some water‐excluding polymers to sample, and subsequently force the precipitation of small EVs out, its time‐saving and easy usage make it suitable for clinical use, though show unavoidable contamination of the isolated EVs with proteins, protein complexes, lipoproteins and nucleoproteins, as well as viral and other particles.^35^, ^36^ There are several polymer kits available that have already applied for purifying the EVs, such as hydrophilic polymers, protamine, sodium acetate and proteins with organic solvent (PROSPR) with along their disadvantages and advantages demonstrating in Table 2.

## 7 IMMUNOAFFINITY ISOLATION METHODS

Immunoaffinity isolation is another approach to isolate the EVs with increasing purity, owing to selectively exploiting the presence of specific molecules in the small EV surface^37^; for example, the lipids, proteins and polysaccharides are common substances that exposed on the surface of EVs, as a result, showing potency in being ligands for manifold molecules. Generally, there are five main methods for the isolation of EVs based on immunoaffinity, including antibodies to EV receptors,^38^ phosphatidylserine‐binding proteins,^39^ heparin‐modified sorbents^40^ and binding of heat shock proteins,^34^ as well as lectins.^41^ Although along with evident advantages of the EV purified isolation, the expensive costs, and the insufficient efficiency of isolation, and difficulties encountering in the process of isolation the large volumes of EVs, which substantially limits the applicability of immunoaffinity isolation methods.

## 8 MICROFLUIDIC DEVICES

Microfluidic devices are composed of a network of microchannels with different sizes, which have been implicated for EV isolation from cell culture and various tissue fluids on the basis of the immunoaffinity principle, as well as systems. However, some issues are yet to be removed; for instance, the inputted sample shows great possibility to block channels and the efficacy of isolation of EVs is extremely slow, consequently, decreasing their diagnostic potential,^38^ and overcome some of the challenges involving in EVs detection, such as the problem of the small size and lacking in distinct biomarkers, which contributing to get a comprehensive understanding function of their contents (eg protein, RNA and lipid).

Several qualitative and quantitative analysis techniques are currently available (Table 3). For instance, transmission electron microscopy (TEM) could be combined with immunogold staining to represent structural details and delineate the subpopulations of EVs.^42^ A study indicates that cryo‐electron microscopy might be more suitable for depicting the morphology of EVs as its no fixation or staining.^43^ The size, morphology and intactness of EVs also could be determined by scanning electron microscopy (SEM) and atomic force microscopy (AFM).^44^ Measuring the size and number distribution of single EVs can be made by dynamic light scattering (DLS) and nanoparticle tracking analysis (NTA).^45^ Both the conventional flow cytometry and novel fluorescence‐based flow cytometry could be promising tools to qualitatively and quantitatively analyse the EVs.^46^ Western blot, enzyme‐linked immunosorbent assay (ELISA) and EVs arrays are used to present purity and enrichment. Micronuclear magnetic resonance [μNMR] system and a photosensitizer‐bead detection system (ExoScreen) are other sensitive qualitative and quantitative approaches.^47^, ^48^, ^49^

**Table 3:** Techniques for extracellular vesicle detection and characterization

Methods	Size detection range/detection limit	Size distribution	Concentration	Marker detection
Quantitative methods				
DLS	1 nm‐6 μm	+	−	−
qNano	70 nm‐10 μm	+	+	−
Qualitative methods				
Western blot and ELISA	NA	−	−	+
Extracellular vesicle array	NA	−	−	+
TEM	<1 nm	+	−	+
SEM	~1 nm	+	−	+
Cryo‐EM	<1 nm	+	−	+
AFM	<1 nm	+	−	−
Quantitative and qualitative methods				
NTA	50 nm‐1 μm	+	+	+
Conventional flow cytometry	≥300 nm	−	+	+
	<300 nm	−	−	+
TRPS	70 nm‐10 μm	+	+	−
Fluorescence high‑resolution flow cytometry	~100 nm	−	+	+
μNMR system	50‐150 nm	−	+	+
nPLEX assay	NA	−	+	+
ExoScreen	NA	−	+	+

Note

“+” indicates variable can be measured, “−” indicates it cannot.

Abbreviations: AFM, atomic force microscopy; Cryo‐EM, Cryo‐electron microscopy; DLS, dynamic light scattering; nPLEX, nanoplasmonic exosome; NTA, nanoparticle tracking analysis; SEM, scanning electron microscopy; TEM, transmission electron microscopy; TRPS, Tunable resistive pulse sensing; μNMR, micronuclear magnetic resonance.

As mentioned above (Table 2), each type of isolation approach has intrinsic advantages and restrictions with respect to cost‐efficacy, complexity, purity, yield and functionality of the EVs. In urological neoplasms similarly, one of the current challenges is how to develop the sensitive detection platforms and robust and remarkable isolation techniques, with promising potency in the identification of EVs and their subpopulations to trigger reliable prognosis and precise prediction of treatment response, or to provide novel neoplasm grading and staging via analysing of easier accessible and minimal‐invasive body fluids. Therefore, many standards should be established to develop such interesting EVs isolation and capture tools. Initially, it is highlighted that the optimal specimen pools should be determined, such as give priority to the sample safety and accessibility, target EVs quantity, and simple and convenient manoeuvrability, especially urine for urogenital tumours; second, the preservation conditions such as temperature, time and additives should be standardized because the quantity and variation of EVs could diminish differential preservation conditions, consequentially giving rise to the deflection of results but also difficulty in continued supervision of cancer development; and third, promising isolation and detection techniques that satisfy the clinical application in a hospital setting should be established.

## 9 GENERAL FUNCTIONS OF EVS IN MALIGNANCIES

**Figure 3:**
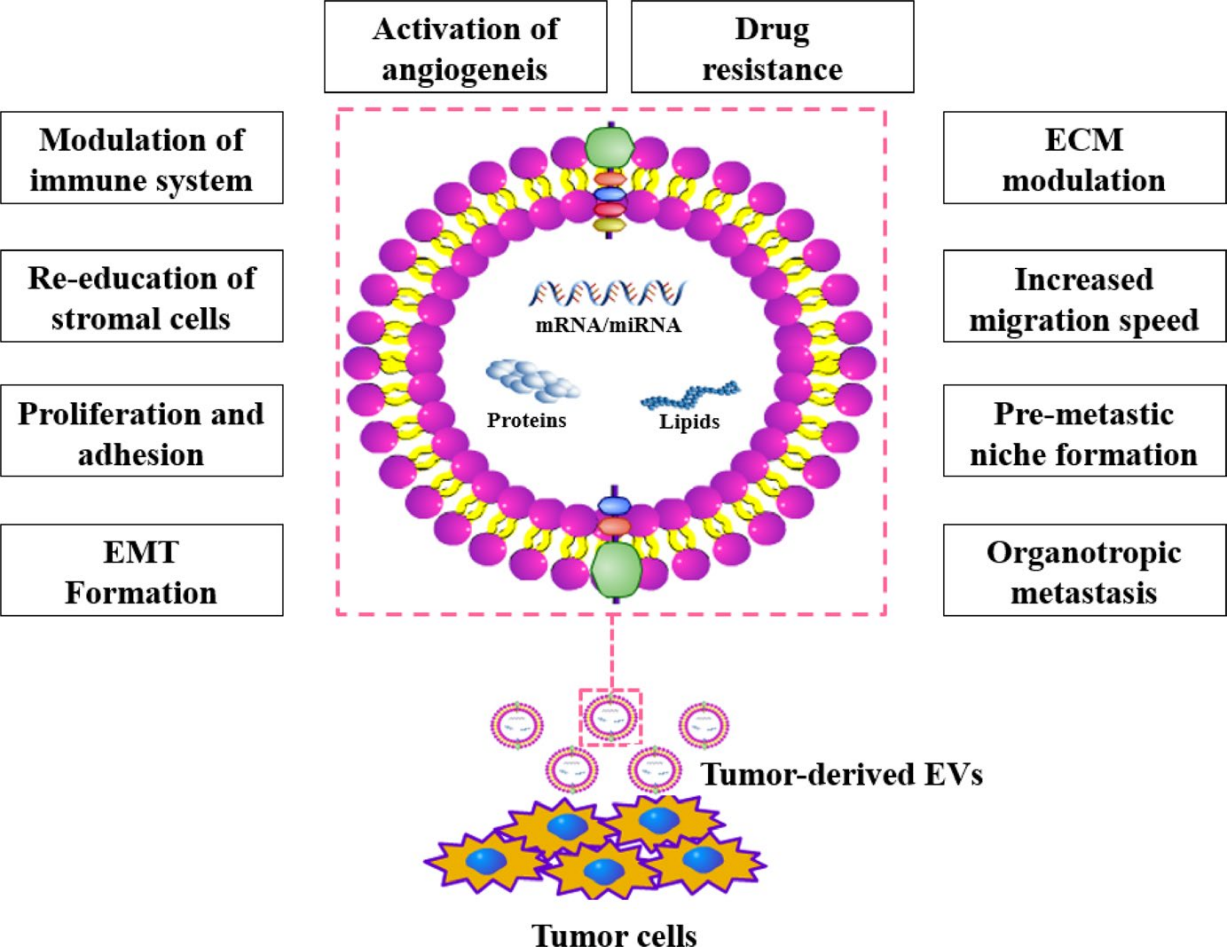
Physiological processes influenced by EVs. Extracellular vesicles are involved in most physiological processes that are associated with intercellular communication, and the content of extracellular vesicles, including mRNAs, microRNAs (miRNAs), lipids and proteins, is depicted

Bioactive molecules of EVs secreted by both cancer cells and tumour‐associated cells provide the essential signals for favouring tumour growth via remodelling the architectures in tumour microenvironments and forming pre‐metastatic niches. Different mechanisms of EVs‐mediated tumour proliferation and progression will be discussed in the following sections (Figure 3).

## 10 PROMOTION OF ANGIOGENESIS

Tumour progression is a dynamic and multistep process requiring continuous nutrient and oxygen supplied by sufficient blood conducts, while also serving to remove waste materials. The advent of cancer stem cells (CSCs) has provided a novel mechanism for the development and progression of the tumour via differentiating into endothelial cells to contribute to the angiogenesis.^50^, ^51^ In addition, a research indicates, for example, that miRNAs, secreted from exosomes, regulate transcription, proliferation, metabolic processing and mRNAs encode vascular endothelial growth factor (VEGF), fibroblast growth factor (FGF), angiopoietin1, Ephrin A3, matrix metalloproteinase‐2 (MMP‐2), and MMP‐9 and growth factors in contrast to CD105‐negative CSCs.^19^

## 11 EPITHELIAL‐TO‐MESENCHYMAL TRANSITION

Epithelial‐to‐mesenchymal transition (EMT) is a developmentally vital reversible process of which fully differentiated cells lose their epithelial features (eg E‐cadherin, β‐catenin and plakoglobin), acquiring a migratory mesenchymal phenotype (eg N‐cadherin and vimentin). EMT also contributes to the metastatic potential of tumours.^52^ Exosome mediates that growth in migration and invasion by the way of EMT has been observed in many other studies.^53^, ^54^, ^55^ Urothelial cells exhibit the EMT after exposing to tumour‐derived exosomes.^56^

## 12 FORMATION OF PRE‐METASTATIC NICHES

Primary tumours can release some biological factors that migrate to preferred metastatic regions and dynamically remodel these sites before spreading to a distant organ, which means that form predetermined metastatic microenvironments, also referred to as pre‐metastatic niches.^57^ In general, exosomes display the characteristics of organ tropism, and the process of the construction of pre‐metastatic niche involves with initial tumour‐derived exosomes releasing into the circulation system and then escaping from the vascular beds to migrate to distant secondary organ.^58^, ^59^ During the process, the crucial initial step is how vascular leaking exosomes can target organ tissues; nowadays, it is induced by complicated processes involved combination of stromal cells and released cancer cell‐derived exosomes resulting in reprogramming of these cells^60^, ^61^, ^62^, ^63^ and activation of several vital signalling pathways,^64^, ^65^ which alter the local chemokine repertoire of the tumour microenvironment (TME) and remodel the components of the extracellular matrix (ECM) in turns.^61^, ^62^, ^66^ Moreover, the vast researches indicate that there are exerting cooperations between bone marrow‐derived cells and exosomes in the primary phase of pre‐metastatic niche that can stimulate the mobilization of these cells into the circulatory system circulation and disseminate to distant sites, sequentially generate a local pro‐inflammatory focus with pro‐tumorigenic immunosuppression.^60^, ^61^, ^62^

## 13 MODULATION OF THE TUMOUR MICROENVIRONMENT

Accumulating evidence support tumour progression is the consequence of communication between tumour cells and cells within the tumour microenvironment via paracrine or autocrine, such as adipocytes, fibroblasts, immune cells and cells of the vascular. It has been shown that tumour cells have a higher propensity to secrete larger quantity of exosome^67^, ^68^; for instance, cancer‐associated fibroblasts (CAFs) support tumour cells in proliferation, delaying senescence and resisting against drugs, which induced by exosome‐secreted miR‐9 and the human telomerase reverse transcriptase (hTERT).^69^, ^70^ Activation of signalling pathways by exosomes is one of the way to modulate the tumour microenvironment, such as irritation of the TGF‐β/Smad pathway by transferring TGF‐β to human umbilical cord‐derived mesenchymal cells (hucMSCs), subsequently differentiating into CAFs.^71^ Moreover, recent research shows that transferring TGF‐β also contributes fibroblasts converse to myofibroblasts, which could secrete insulin‐like growth factor 1 (IGF‐1), activin A and VEGF to induce tumour progression.^72^ The irritation of another signalling pathway by exosomes in the bidirectional crosstalk between cancer cells and normal stromal cells, such as nuclear factor kappa B (NF‐κB) and epidermal growth factor receptor (EGFR) signalling, also plays vital roles in the proliferation and migration of tumour. Endothelial cells show enhanced cell motility and tube formation ability after re‐educated by tumour‐derived exosomes^73^; moreover, RNA secreted from EVs develops hepatocyte growth factor synthesis through the activation of ERK1/2 and AKT signalling pathways.^74^ It is widely believed that tumour‐derived EVs impose significant effects in mediating communication between immune and cancer cells of renal cell cancer (RCC),^75^ such as immune evasion of tumours.^76^, ^77^ MiR‐222‐3p induces polarization of tumour‐associated macrophages by the activation of SOCS3/STAT3 pathway to facilitate tumourigenesis and cancer progression.^78^ Additionally, Rab27a supports exosome could modify the tumour microenvironment via advancing recruitment and differentiation of bone marrow‐derived neutrophils to cancer cells.^79^ Furthermore, a few studies suggest that tumour‐derived EVs facilitate cancer progression by attenuating immune and more specific EVs could diminish the cytotoxicity of natural killer cells and T cell in immunoreaction.^80^, ^81^, ^82^ Tumour‐derived EVs also could influence the cancer cells themselves via autocrine to irritate the invasion and migration, and reduce adhesion abilities as well via enhancing MMP‐9 or chemokine receptor type 4 (CXCR4).^74^

## 14 MANAGEMENT OF UROLOGIC MALIGNANCIES

In the past several decades, although with the increasing development of renewable treatments for urological tumours, for instance chemotherapies and molecular targeted therapies and renowned immunotherapies, the prognosis remains poor. Recently, sufficient researches on EVs in urinary tumours provide a deeper understanding of biogenesis and pathogenesis and might be offered underlying therapeutic targets in urologic cancers. Herein, we review the current published research on EV for commonly urogenital carcinomas including bladder cancer (BCa), kidney cancer (RCC) and prostate cancer (PCa).

## 15 KIDNEY CANCER (RENAL CELL CANCER)

Renal cell cancers (RCCs) represent 2%‐3% of all cancers.^83^, ^84^ Many kidney cancers remain asymptomatic until the late disease stages with 50% of patients are detected incidentally by non‐invasive imaging investigating, and approximately 30% of patients with metastasis in the primary time of diagnosis.^85^, ^86^ The utility of urinary EVs could be recognized as potential diagnostic and prognostic markers in RCCs (Table 4).^87^ Proteomic analysis of urinary EVs differs from the patients and healthy groups with showing an RCC‐specific signature of the effectiveness of proteins.^58^, ^88^ Another proteins, for instance, podocalyxin (PODXL), WNT signalling pathway inhibitor 4 (DKK4), ceruloplasmin, carbonic anhydrase IX (CAIX) and MMP‐9, were validated by using immunoblotting method.^89^ In addition, among RCC patients, the mRNA levels of glutathione S‐transferase alpha 1 (GSTA1), CCAAT enhancer binding protein alpha (CEBPA) and PCBD1 in EVs decrease compared with healthy groups.^90^ Moreover, a report demonstrates that the lipids within urinary EV show difference between RCC patients and healthy groups.^91^ Based on microRNA expression screening, the cluster of miRNAs including miR‐449a, miR‐126‐3p, miR‐486‐5p and miR‐34b‐5p could differentiate clear cell RCCs from benign subjects^92^ and could be recognized as potential diagnostic and prognostic markers.^58^, ^93^, ^94^ Serum‐derived EVs have also been recognized as prominently diagnostic and prognostic tools for clear cell RCCs.^93^, ^94^ Recently, azurocidin as a permeabilizer for vascular endothelial cells has been isolated from serum‐free medium within incubating clear cell RCC tissues samples.^95^

**Table 4:** Candidate biomarkers for kidney cancer derived from EVs

Source	Methodologies	End point	Type of marker	Markers	Reference
Urine	Ultracentrifugation	Diagnosis	mRNA	GSTA1, CEBPA and PCBD1	De Palma et al^89^
Urine	Density gradient ultracentrifugation	Diagnosis	Proteins	MMP‐9, PODXL, DKK4, CAIX and ceruloplasmin	Raimondo et al^88^
Cancer stem cells	Ultracentrifugation, Flow cytometry immunohistochemistry	Diagnosis	Proteins	VEGF, FGF, angiopoietin 1, Ephrin‑A3, MMP‑2, MMP‑9	Grange et al^57^
RCC cells	Centrifugation Filtration Flow cytometry Western blot ELISA	Diagnosis	Proteins	Fas ligand, Bcl2‑L‑4	Yang et al^87^
Viable human tissue	Ultracentrifugation Mass spectrometry Western blot	Diagnosis	Proteins	Azurocidin 1	Jingushi et al^93^
Serum	Immunoaffinity magnetic beads	Diagnosis	miRNA	miR‐210 and miR‐1233	Zhang et al^92^
Serum	Total Exosome isolation kit	Prognosis	miRNA	miR‐224	Fujii et al^93^
Cancer stem cells	Ultracentrifugation Microarray analysis qRT‑PCR	Diagnosis	miRNA	miR‑200c, miR‑92, miR‑141, miR‑19b, miR‑29a, miR‑29c, miR‑650, miR‑151	Grange et al^94^
Urine	Centrifugation Urine exosome RNA isolation kit	Diagnosis	miRNA	miR‐126‐3p, miR‐449a, miR‐34b‐5p, miR‐486‐5p	Butz et al^91^
Urine	Ultracentrifugation Mass spectrometry	Diagnosis	Lipids	Lysophosphatidylethanolamine metabolite	Del Boccio et al^90^

Renal cell cancers generate EVs that could educate endothelial^95^ and immune cells,^96^ with promoting angiogenesis and immunosuppressive activity, respectively. As for immune systems, increasing evidence shows that tumour‐derived EVs result in immune evasion of tumours partly via the activation of caspase pathway to trigger apoptosis in T lymphocytes.^97^ In addition, both EVs‐derived antigens and Hsp70 can inhabit the immunoreaction though induction tumour growth factors of and pro‐inflammatory cytokines similarly.^98^, ^99^ Moreover, EVs also can be applied for cancer immunotherapy, mainly due to promoting cytotoxic effects and proliferation of T cells via releasing interferons.^100^ Furthermore, RCCs also could secrete EVs to interact with endothelial cells to promote lymphopoiesis and angiogenesis and thereby metastasis. A study shows that EVs derived from RCC cell line (eg 786‐O) increase the expression of vascular endothelial growth factor in human umbilical vein endothelial cells (HUVECs) with resulting in tubular formation of HUVECs,^97^ involving the downregulation of hepatocyte cell adhesion molecule by upregulating phosphorylated AKT in RCCs.^101^ What is more, CD105^+^ stem cells of RCC release EVs that promote and trigger the formation of a pre‐metastatic niche by upregulating MMP2 and VEGF^58^, ^102^; although sunitinib is a first‐line targeted regimen for metastatic RCCs,^103^ its efficacy of biotherapy for the long time is controversial in respect to drug resistance, which has been confirmed that regulated by lncARSR by promoting MET and AXL expression in RCC cells.^104^, ^105^ Therefore, all those support that EV‐based targets display a promising potency for the development of novel cancer therapies.

EVs also can be served as a vaccine for RCCs. A report reveals that RCCs derived from EVs could increase immunogenicity by proliferating T cells and releasing interferons subsequently^100^; the effectiveness of vaccines when dendritic cells (DCs) load with EVs is higher rather than whole tumour lysate.^98^

## 16 THE BLADDER CANCER

Bladder cancer (BCa) is the seventh most commonly diagnosed cancer in the male population worldwide, and the diagnosis for BCa is usually on the basis of cytology, urinalysis and cystoscopy. Cytology is a highly specific test, but low in the sensitivity for the diagnosis of BCa.^106^ Cystoscopy is the gold standard to diagnose the BCa, while this method is expensive and invasive, even for flexible cystoscopy, and the risk of developing urinary infections is up to 10%^107^; non‐invasive and reliable biomarkers are therefore required in the future. Given that, urine is an excellently suitable fluid for biomarkers discovery in BCa. The biomarkers (mainly including proteins, mRNA, lncRNA and miRNA) within EVs isolated from BCa were investigated by different research groups, which could be promising molecules to identify the BCa and predict the progression of the BCa (Table 5). Based on proteomic analysis of urinary EVs, several studies have been identified cargoes of possible biomarkers for BC patient, but not in healthy volunteers.^108^, ^109^ Among them, research shows that the levels of tumour‐associated calcium‐signal transducer 2 (TACSTD2) are correlative with BCa, compared with high‐grade BCa. The author identified seven proteins differentially expressed in the low‐risk group (Table 5).^109^ Proteome profiling of urinary exosomes indicates H2B1K and alpha 1‐antitrypsin as prognostic and diagnostic biomarkers for urothelial bladder cancer, which could be verified in immunohistochemistry (IHC).^110^ Additionally, HEXB, S100A4 and SND1 significantly identified in EV derived from the MIBC cell line also are upregulating in urinary EV from MIBC patients when vs to normal groups.^111^ There are other proteins could be recognized as potential diagnostic and prognostic markers for BCa.^112^, ^113^, ^114^, ^115^ Using a whole transcriptome array, a study reports the potential application of mRNAs in urinary EVs for diagnoses, such as LASS2 and GALT1 involving progression with only in BCa patients, and ARHGEF39 and FOXO3 while only expressing in healthy controls to suppress the tumour.^116^ In addition, based on the microarray analysis of miRNA, great studies pay their attention on the roles of diagnostic and prognostic.^117^, ^118^, ^119^, ^120^, ^121^ Interestingly, research shows that several microRNAs from urinary EVs significantly upregulate in BCa, but not in plasma from same patients, which suggests that different biofluids may harbour different molecules.^122^

**Table 5:** Candidate biomarkers for bladder cancer derived from EVs

Source	Methodologies	End point	Type of marker	Markers	Reference
BCC/urine	Ultracentrifugation Flow cytometry In‑gel digestion Mass spectrometry	Diagnosis	Proteins	β1 and α6 integrins, CD36, CD44, CD73, CD10, MUC1, basigin, 5T4	Welton et al^107^
Urine	Ultracentrifugation	Diagnosis	Proteins	APOA1, CD5L, FGA, FGB, FGG, HPR, HP	Chen et al^108^
Urine	Differential ultracentrifugation	Diagnosis Prognosis	Proteins	Alpha‐1 antitrypsin, histone H2B1K	Lin et al^109^
Urine	Differential ultracentrifugation	Diagnosis	Proteins	HEXB, S100A4, SND1	Silvers et al^110^
BCC/urine	Sucrose/D2O cushion Ultracentrifugation	Diagnosis	Proteins	EDIL‐3	Beckham et al^111^
Urine	Ultracentrifugation In‑gel digestion Mass spectrometry	Diagnosis	Proteins	Resistin, GTPase NRas, MUC4, EPS8L1, EPS8L2, EHD4, G3BP, RAI3, GSA	Smalley et al^112^
Urine	Centrifugation Filtration Integrated double‐filtration Microfluidic device	Prognosis	Proteins	CD63 + EV signal intensity	Liang et al^113^
Urine	Ultracentrifugation	Prognosis	Proteins	Periostin	Silvers et al^114^
Urine	Ultracentrifugation NanoSight microarray PCR	Diagnosis	mRNA	LASS2, GALNT1	Perez et al^115^
Urine	Differential ultracentrifugation	Diagnosis	miRNA	miR‐21‐5p	Matsuzaki et al^116^
Urine	Differential ultracentrifugation Filtration	Diagnosis Prognosis	miRNA proteins	miR‐375, miR‐146a, apoB	Andreu et al^117^
Urine	Differential centrifugation Total exosome isolation kit	Prognosis	miRNA	miR‐141‐3p, miR‐200a‐3p, miR‐205‐5p	Baumgart et al^118^
Urine	Differential ultracentrifugation	Prognosis	miRNA	miR‐940	Long et al^119^
Urine	Nanostring miRNA assays Droplet digital PCR	Diagnosis	miRNA	miR‐205, miR‐200c‐3p, miR‐29b‐3p; miR‐921, miR‐23b	Ostenfeld et al^120^ Berrondo et al^122^
Urine	Centrifugation Exosome RNA isolation kit	Diagnosis	miRNA	miR‐4454, miR‐21, miR‐720	Armstrong et al^121^
Urine	Differential ultracentrifugation	Diagnosis Prognosis	mRNA; lncRNA	HOTAIR, HOX‐AS‐2, MALAT1, SOX2, OCT4, HYMA1, LINC00477, LOC 100506688, OTX2‐AS1	Berrondo et al^122^

Furthermore, to unravel the roles of lncRNA in BCa, recent researches show that lncRNAs, such as HOX‐AS‐2, HOTAIR, ANRIL and lnc‐RoR, are enriched in BCs cancer cell line EVs as well as urinary EVs from high‐grade BCa patients, demonstrating that lncRNAs have potential as biomarkers for BCa.^123^ Additionally, recent reports demonstrate that the CD63^+^ urinary EVs could be a biomarker for the detection of BCa.^114^, ^124^ EVs derived from BCa cell line also influence local regions microenvironments or distant cells by transferring their content, as result of facilitating proliferation, angiogenesis, invasion, and migration and the inhibition of apoptosis^56^, ^112^, ^125^; for example, EGF‐like repeat and discoidin I‐like domain‐containing protein 3 (EDIL‑3) from invasive BCa cell lines could stimulate the migration and angiogenesis of urothelial and endothelial cells.^112^ Periostin, another factor from invasive BCa cell lines, could contribute low‐grade BCa cancer cells to gain the aggressiveness within via activating ERK oncogenic pathway.^115^ Urothelial cells exposed to EVs from cancer cell lines or patient specimen show the phenomena of EMT.^56^ Similarly, lncRNA‐UCA1, which may be play an important role in causing intratumoural hypoxia, could irritate tumour progression via the EMT as well.^126^ However, exosomes also can discard tumour‐suppressive miRNAs contributed to BCa progression, such as miR‐23b and miR‐921,^121^ all those could provide underlying targets for the future therapies for the BCa. Recently, a report demonstrates the effectiveness of EVs as a vector to carry siRNAs in BCa; therefore, in the future, EVs have the potential functions to stably deliver substantial therapeutic cargoes including miRNAs and siRNAs, to anchor organs with the development of tissue engineering technology.^127^

## 17 PROSTATE CANCER

Prostate cancer (PCa) is the second most commonly diagnosed cancer in men, accounting for 15% of all cancers diagnosed.^128^ Although PSA testing contributes to identify and manage PCa in the early phase, it still has some limitations, for example, the specificity of discrimination of benign prostate diseases, such as acute prostatitis and benign hyperplasia.^129^ Thus, the more specific and ideal substrate (eg urine, prostatic plasmas and blood samples) for PCa are urgently developed rather than invasive prostate biopsies.^130^ Some studies have presented the usefulness of urinary EVs as diagnostic factors (Table 6).^131^, ^132^, ^133^, ^134^ EV‐involved transmembrane proteins CD63 and CD9 are sufficient in urine from PCa.^135^ Integrin α3, δ‐Catenin, integrin β1 and FABP5 proteins are identified in urinary EVs of PC patients with the significantly increased levels of PCa patients during the process of the investigation of the proteomic cargo of urinary PC‐derived EV.^136^, ^137^, ^138^, ^139^ Moreover, on the basis of the mass spectrometry proteome analysis, thousands of proteins encapsulated on and within vesicles are identified as biomarker candidates from urinary EVs or cell lines of PCa,^139^, ^140^, ^141^, ^142^, ^143^, ^144^, ^145^, ^146^ whereas the value as biomarkers is still controversial, and several types of research regenerate the previous biomarkers by the using targeted proteomics and immuno‐assays.^134^ Based on a proximity ligation assay, PC‐derived EVs in blood has also shown to contain proteins specific to PCa, such as phosphatase and tensin homolog gene (PTEN), survivin and other factors with decreased level compared to benign prostatic hyperplasia or health subjects.^133^, ^147^, ^148^, ^149^ PC‐based EVs show great value in comprehensively mapping nucleic acid changes in PCa via urine‐ or blood‐based liquid biopsies, and it has been known that the EVs is important pool resource for circulating‐free DNA (cfDNA)^150^; as such, some factors demonstrated good clinical usefulness and diagnostic value in predicting for high‐grade PC, including TP53 mutations, ERG and PCA3, can be detected in the EVs.^150^ ExoDx Prostate IntelliScore urine exosome assay has been developed as a non‐invasive detective tool to distinguish the high‐grade PCa from low‐grade groups and benign diseases at initial biopsy.^151^ Several mRNAs could be recognized as promising diagnostic and prognostic tools for the PCa^152^, ^153^, ^154^, ^155^, ^156^; for instance, the transcripts of CDH3 from EVs were significantly decreased compared with benign hyperplasia^156^; contrarily, the mRNA level of PTEN gene can only be detected in the patient of PC,^147^ and nevertheless, both of them are expected to be powerful for the diagnosis and monitoring of PCa.^152^ RT‐PCR, microarray and RNA sequencing technologies have focused on the non‐coding RNA content within EVs so far; for example, in the urinary sample, the levels of lncRNA‐p21, a suppressor of p53 signalling, contribute to detecting PC from benign disease.^157^ Next‐generation sequencing reveals the potential values for miRNA served as diagnostic and prognostic biomarkers for PCa within serum or plasma EVs,^41^, ^131^, ^132^, ^158^, ^159^, ^160^, ^161^, ^162^ such as miR‐141 and miR‐375 in serum, have been correlated with metastatic PCa.^159^, ^163^ Another study indicates that exosomal miR‐1290 and miR‐375 could be as prognostic markers in castration‐resistant prostate cancer (CRPC).^164^ In recent, several research works demonstrate that the lipids including diacylglycerol and triacylglycerol are differentially enriched in PCa rather than healthy groups.^165^, ^166^ Glycomic and metabolomic profiling of urinary EV reveal several small molecule metabolites could be novel biomarkers to predict the development of PCa, for example levels of N‐linked glycans, glucuronate, adenosine, d‐ribose‐5‐phosphate and isobutyryl‐l‐carnitine.^167^, ^168^

**Table 6:** Candidate biomarkers for prostate cancer derived from EVs

Source	Methodologies	End point	Type of marker	Markers	Reference
Plasma	Ultracentrifugation Western blot ELISA	Diagnosis	Proteins	Survivin	Khan et al^132^
Urine	Differential ultracentrifugation Filtration	Diagnosis Prognosis	Proteins	TGM4, ADSV, PPAP, PSA, CD63, SPHM, GLPK5 TMEM256, flotillin 2, Rab3B, PARK7, LAMTOR1 TM256, LAMTOR1, ADIRF TMEM256, flotillin 2, Rab3B, PARK7, LAMTOR1	Wang et al^133^ Sequeiros et al^145^
Urine	Differential ultracentrifugation	Diagnosis Prognosis	Proteins	δ‑catenin Integrin α3, Integrin β1 FABP5	Liu et al^135^ Lu et al^136^ Fujita et al^138^
Tissue Urine	Differential ultracentrifugation Filtration	Diagnosis Prognosis	Proteins	CD63, ANXA1‐3, FASN, FOLH1, GDF15, MDR1, XPO1, TGM4, TIMP1, SFN, TMEM256, LAMTOR1, ADIRF, ITGA3, and ITGB1	Bijnsdorp et al^137^
Tissue	Ultracentrifugation Gel filtration Chromatography, 2D‐PAGE Mass spectrometry	Diagnosis Prognosis	Proteins	ANXA1, ANXA3, ANXA5, DDAH1	Ronquist et al^140^
Cell lines	Ultracentrifugation Mass spectrometry Bead immuno‑isolation Western blot	Diagnosis Prognosis	Proteins	CDCP1, CD151, CD147	Sandvig et al^141^
Cell lines Urine	Ultracentrifugation immunoprecipitation Western blot Electron microscopy	Diagnosis Prognosis	Proteins	ANXA2, CLSTN1, FASN, FLNC, FOLH1, GDF15ACPP, LTF, DDP4, TGM4, MME, PSA, SEMG1, AZGP1, ANPEP, G3BP, PSMA, TMPRSS2, FASN, LGALS3, PSCA, KLK2, KLK11, TIMP1 PDCD6IP, XPO‑1, ENO1	Duijvesz et al^142^ Utleg et al^143^ Principe et al^144^
Plasma	Ultracentrifugation Western blot immunofluorescence	Diagnosis	Proteins	PTEN	Gabriel et al^134^
Serum	Differential centrifugation	Predictive Monitoring	Proteins	ABCB1, ABCB4, PABPC4	Kato et al^147^
Urine Plasma	Differential ultracentrifugation Filtration Chromatography	Diagnosis	Proteins	Afamin, cardiotrophin‐1, CDON, endoplasmic reticulum aminopeptidase 1, FGF19, IL17RC, NAMPT, IL1RAPL2, CD226, IGFBP2, CCL16, TNFSF18, IGFBP5; Aromatic‐l‐amino‐acid decarboxylase	Welton et al^148^
Urine	Centrifugation Filtration Ultrafiltration	Diagnosis Prognosis	mRNA	PCA3, TMPRSS2‐ERG AGR2, SV‐G, AGR2 SV‐H CDH3	Neeb et al^151^ Donovan et al^152^ Hendriks et al^153^ Motamedinia et al^154^ Royo et al^155^
Plasma/Serum/Urine	ExoMiR extraction Filtration qRT‑PCR	Diagnosis	miRNA	miR‑107, miR‑130b, miR‑181a‑2, miR141, miR‑301a, miR‑326, miR‑331‑3p, miR‑375, miR‑432, miR‑574‑3p, miR‑22110, miR‑625 miR‐1290, miR‐375, miR‐574‐3p, miR‐141‐5p, and miR‐21‐5p miRNA‐21, let‐7c, miR‐196a‐5p, miR‐501‐3p, miR‐19b, miR‐145	Samsonov et al^41^ Foj et al^130^ Bryant et al^158^ Bryzgunova et al^159^ Rodríguez et al^131^ Wani et al^160^ Xu et al^161^
Urine	Differential centrifugation Urine exosome RNA isolation kit	Diagnosis	lincRNA	lincRNA‐p21	Işin et al^156^
Urine	Differential ultracentrifugation Filtration	Diagnosis	Lipids	Lactosylceramide, phosphatidylserine, phosphatidylglycerol, diacylglycerol, triacylglycerol	Skotland et al^164^ Yang et al^165^
Urine	Differential ultracentrifugation Filtration	Diagnosis	Metabolites	Adenosine, glucuronate, isobutyryl‐l‐carnitine, d‐ribose 5‐phosphate	Puhka et al^166^
Urine	Differential ultracentrifugation	Diagnosis Prognosis	Glycomic	N‐linked glycans	Nyalwidhe et al^167^

The intercellular crosstalk through EVs could stimulate tumour progression. Several proteins presenting on and in EVs from PCa cell lines are recognized as significant meditators for the biological communication between cancer cells and tumour microenvironment or surrounding cell, including cytokine CX3CL1, MMPs and transforming growth factor B, play significant roles in the proliferation and differentiation of fibroblasts.^169^, ^170^ In addition, integrins ITGA3 and ITGB1 can affect invasion and migration of normal prostate epithelial cells.^138^ Several studies suggest that complicate intercellular interactions between cancer cells, osteoclasts and osteoblasts contribute to bone metastasis.^171^, ^172^ It is the first protein that has been reported in the EVs originated from human hormone‐refractory PCa cells to facilitate mouse pre‐osteoblast differentiation.^171^ Another molecular miR‐141‐3p is involved with the osteoblastic metastasis of prostate cancer via reducing the expression of Deleted in Liver Cancer 1 (DLC1) and activating the p38MAPK and OPG/RANKL pathway in osteoblasts^173^; however, another research fixes their attention on the role of EVs derived from PCa cell line in the osteoclast for the bone metastasis promoting osteoblast proliferation and identifies that tumour cell‐derived EVs play important roles in impairing the osteoclast formation and differentiation through the underlying mechanism is unknown yet.^172^ Moreover, the effect of biological crosstalk between PCa cell and immune cells though EVs causes the induction immune suppression via down‐regulating the NKG2D cytotoxicity receptor and diminishing the IL‐2 response.^174^, ^175^

PCa‐derived EVs also involve drug resistance. ABCB1, ABCB4, PABPC4 and SH3GL1 are much more sufficient in EVs from docetaxel‐resistant prostate cancer cell lines and potentially higher in serum EVs in men with docetaxel‐resistant PCa.^148^ AR‐V7 is correlated with resistance to enzalutamide and abiraterone in metastatic CRPC patients, which could be a biomarker to predict the CRPC.^176^, ^177^ Finally, EVs‐derived PCs can be used as vaccine vesicles that present prostate‐associated antigens such as PSA and PAP on their membrane to exert an anti‐tumour immune response.^98^, ^178^, ^179^

## 18 DISCUSSION

**Figure 4:**
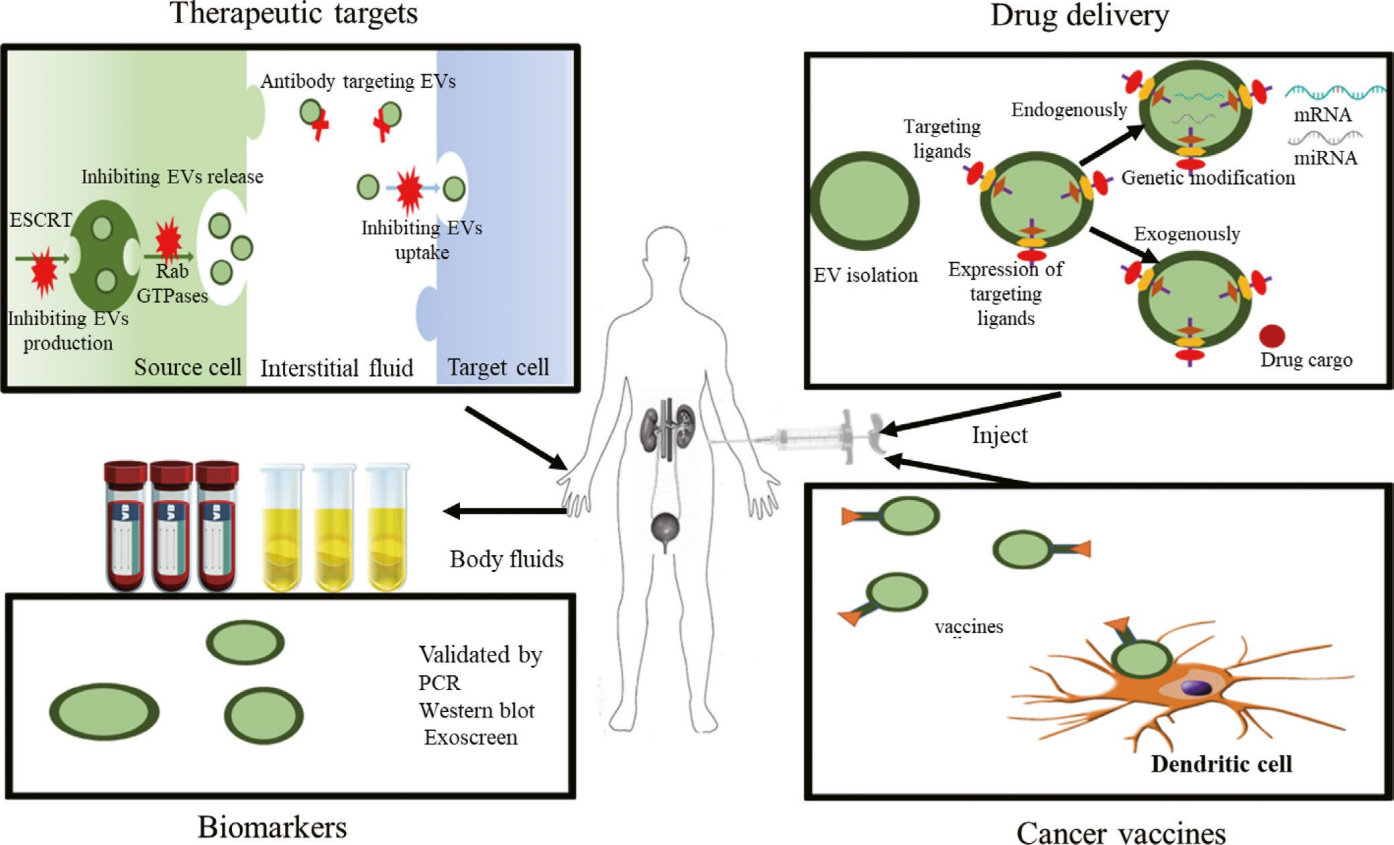
Future implements for EVs in urological cancer. EVs impact the multistep process of cancer; therefore, EVs should be a novel treatment strategy by inhibiting intercellular crosstalk. EVs could serve as promising diagnostic and prognostic biomarkers to dynamically trace the changes in cancer due to their high specificity and sensitivity. In addition, EVs have the potential functions to stably deliver substantial therapeutic cargoes liking miRNAs and siRNAs with stability, few side effects and organ specificity. Furthermore, several studies have reported the potential of EVs derived from dendritic cells used as vaccine vesicles. Copyright 2018, The Jikei University School of Medicine, Fumihiko Urabe^19^

With the advent of novel concepts involving EVs in many physiologic as well as pathologic conditions, the field of EV research develops much excitement in the urologic malignancies. Unlike conventional biopsies of that only consist of a small amount of tumour solid masses with ignoring heterogeneity, EVs, as liquid biopsies, could capture and obtain overall tumour heterogeneity owing to directly releasing from all cells in the cancer tissue and its microenvironment. Beyond a doubt, specific bioactive contents contained in circulating EVs have great promise as reliable surrogates of urological cancers; therefore, their molecular cargoes including nucleic acids, protein and lipid composition, as well as their numbers, are representing as diagnostic, prognostic biomarkers for urinary tract diseases, and immense promising for therapeutic advancements. To conclude, we have a faith for the implement of EVs in urological cancers diagnosis and therapeutics owing to their enormous potencies in several aspects, as described below (Figure 4).^19^

## 19 ROLES IN DIAGNOSTIC AND PREDICTIVE BIOMARKERS

As more is understood about the fundamentals of EVs biology and roles involved in tumorigenesis and therapy resistance, EVs‐based analytical methods are increasingly interesting targets for clinical application. EVs are directly released from heterogeneous tumorous and reflect a snapshot of the current state of the neoplasm; therefore, EVs have great potential as remarkable, specific and sensitive biomarkers of oncogenesis, treatment response and therapy resistance. In urogenital cancers, it is thought that increased exosomes are produced by more advanced cancers, and it thus has been suggested that total circulating exosome burden may serve as indicators for disease surveillance. Exosomal contents can also identify disease or predict treatment response, such as several proteins (eg PD‐1, PD‐L1) or some nucleic acids (eg miRNA) with the roles as diagnostic biomarkers for cancer, indicators for therapeutics, worse still, research to date strongly indicate EVs involve treatment irresponsiveness. Such phenomenon was observed in various malignancies also including urogenital cancers; for example, docetaxel‐sensitive cell lines of prostate carcinoma undesirably acquire drug resistance again when co‐culture with EVs derived from the drug‐resistant cells.^180^ To date, increased pumping agents out of tumorous cells or omics alternations induced by the cargo of EVs are two most common opinions, but the underlying mechanisms are still entirely unknown. Further investigations that tailored clinical studies are now warranted to determine how best to prevent this occurring, in the interest of patients and also for economic benefit. Additionally, endothelial cell‐derived EVs can reflect transient cellular stress conditions and could be useful as predictors of anti‐angiogenic therapy effectiveness and cancer cell status. However, issues with interpretation of studies and reproducibility have arisen due to the deficit of standard isolation and characterization, and nomenclature employed, and as the result of the publication of the Minimal Information for Studies of Extracellular Vesicles 2014 (MISEV 2014) with emphasizing that build the set of biophysical, biochemical and functional standard that help in detecting particular biological cargo or functions in extracellular vesicles.^181^ Until 2018, the updated MISEV guidelines were published, and it continued to standardize the experimental parameters for EV isolation and characterization to provide more reproducible and robust outcomes.^182^ Continued efforts to systematically catalogue the protein, nucleic acid and lipid constituents of EVs isolated from richly annotated specimens could ultimately attribute to rapidly evolve and expand the development of selective and sensitive capture platforms directed towards specific EVs.

## 20 ROLES IN THERAPEUTICS

Directly or indirectly, EVs derived from tumour or TME can influence urogenital neoplasm via intercellular crosstalk or modification of TME, respectively. As discussed above, cargoes of tumour‐derived EVs attribute to cancer development. Thus, the blockage of exosome production, secretion and ablation of specific active exosomal contents, as well as exosome‐mediated cell‐cell communication between cancer and TME, have been proposed as alternative therapeutic strategies. Importantly, it is essential to note that EVs show promising potency for immunotherapy. As we all known, immunotherapy have Immunotherapy has revolutionized cancer therapy, especially the advent of immune checkpoint blockades (ie PD‐1/PD‐L1). Recently, it has been demonstrated that cancer‐derived exosomes transfer functional PD‐L1 and inhibit immune responses,^183^ while suppression of exosomal PD‐L1 induces systemic anti‐tumour immunity and memory in urological carcinomas,^184^ and clinical trials have already been initiated to explore their safety and efficacy in humans. Interestingly, EVs‐based vaccines can serve as new candidates that have shown their potential as novel cancer intervention in some clinical trials, indicating that rely on their role as tumour antigens and facilitate an anti‐tumour immunity in turns. The popular EVs‐based vaccines mainly derive from dendritic cell (Dex immunotherapy), but the clinical efficacy is not ideal to date; further research will be required to reassess clinical applications with taking the defects in current prospective designs into considerations such as lack of preselection criteria and small sample size. EVs, on the other hand, have garnered much attention as several characteristics of an optimal delivery system. First of all, the nanometric‐sized EVs confer the effective assimilation and intracellular trafficking for recipient cells. Second, the EV bioactive molecules are protected from degradation in the extracellular milieu and circulation due to lipid bilayer‐membrane structure of EVs.^185^ Third, autologous EVs show lower immunogenicity and toxicity than other conventional drug‐delivery platforms.^186^ Furthermore, EVs possessing specific surface proteins (eg integrins) could bear intrinsic targeting properties that are able to interact with target cells or organs.^187^ Although EVs have many advantages as described above, this enormous promise therapeutic delivery tool requires further study for clinical applications. These include the identification of the optimal EV donor cell type, large‐scale EVs isolation, preservation of EV structural integrity during drug loading, scalable manufacture and storage. A further challenge remains improving methods to shift in vivo biodistribution of EVs from non‐specific sites towards accumulation in desired tissues. Although considerable efforts by engineering EVs to present cell type‐specific ligands have been made in guaranteeing rich accumulation in target tissues, one of the major obstacles remains low delivery efficacy. The elucidation to these questions will enhance rationality and reliability to irritate the utility of EV‐involving molecular cargoes as cancer diagnostics in the clinical practices.

## 21 CONCLUSION

In recent, EVs have gained rocketing interest in the field of urological tumour research owing to their multifaceted role in the development and treatment of cancer, and their perspective as a weapon to the armoury for cancer treatment. Since EVs play pivotal effects on intercellular interactions in variable biological fluids, their numbers, protein, nucleic acids, lipid and signalling/epigenetic regulators components could be transfer to recipient cells and subsequently affect the pathologic process of the receptor cells, eventually result in abnormal proliferation, EMT, angiogenesis and metastasis by regulation of the TME to cause drug resistance, and preparation of pre‐metastatic niches, to enhance dissemination of cancer cells and cause relapse after a prolonged period of dormancy; thereby, targeting this communication will offer a novel therapeutic strategy for urologic cancer eradication. Apart from that, EVs could serve as a non‐invasive liquid biopsy and have been emerged as new potential diagnostic/prognostic biomarkers, as well as playing provoking roles in predicting anti‐cancer drug responses. In additions, we could use EVs as cancer vaccines or as drug delivery modules with promising therapeutic applications, unless more researches are required for clinical applications. However, the research in EVs is encountered with urgent challenges, including the standardization of approaches for the isolation, quantification and analysis of EVs from complicated tissues sample (mainly from cancer line cell medium or low numbers of patient urine samples). Great efforts have been made to precisely determine EV particles and, nevertheless, still have a great way to standardize EVs enumeration in some particular specimens, such as blood. Moreover, a further challenge is what EVs, their contents or their ratio should best be quantified as robust biomarkers in the surveillance of urological diseases staging is yet unknown; thus, in the future studies, we need pay more attention to develop stereospecific antibodies to map the topography of EVs. Furthermore, another big problem in the field is their half‐life in human samples are yet unexplored. With the increasing knowledge of their roles and development of the next‐generation sequencing, mass spectrometry‐based metabolomics and proteomics, we are enthusiastically sure that EVs will contribute to play clinical applications for urological cancer treatment and management in the near future.

## CONFLICT OF INTEREST

The authors declare that they have no competing interests.

## AUTHOR CONTRIBUTIONS

Z.W., S.W. and Z.Z. designed the review and made a retrieval strategy; Z.W. and Y.L. drafted the review text; W.X. and J.C. drafted the tables and figures; and both authors contributed to revision and finalization of the manuscript.

## CONSENT FOR PUBLICATION

The patient has given his consent for his case report to be published.

## Data Availability

Research data are not shared.
